# Children's friendship stability in the United States, China, and Indonesia: Associations with individual attributes and dyadic similarity

**DOI:** 10.1111/cdev.14189

**Published:** 2024-10-26

**Authors:** Luhao Wei, Kristine Marceau, Xinyin Chen, Scott Gest, Junsheng Liu, Dan Li, Doran C. French

**Affiliations:** ^1^ Department of Human Development and Family Science Purdue University West Lafayette Indiana USA; ^2^ Graduate School of Education University of Pennsylvania Philadelphia Pennsylvania USA; ^3^ School of Education and Human Development University of Virginia Charlottesville Virginia USA; ^4^ Department of Psychology East China Normal University Shanghai China; ^5^ Department of Psychology Shanghai Normal University Shanghai China

## Abstract

Predictors of friendship stability from individual attributes and dyadic similarities were assessed using cross‐classified multilevel analyses in this 6‐ to 8‐month longitudinal study of 10‐year‐old US (White, Black, Asian, other; *n* = 477, 50% girls), Chinese (*n* = 467, 59% girls), and Indonesian (Sudanese, Javanese, other; *n* = 419, 45% girls) children with complete participation and reciprocated baseline friendships. Across countries, individual attributes of social preference, popularity, and academic achievement and dyadic social preference similarity positively predicted friendship stability. Dyadic similarity of popularity, academic achievement, and aggression respectively predicted friendship stabilities of US, Chinese, and Indonesian children. Both individual attributes and dyadic similarity predicted friendship stability, with results that varied across countries consistent with attributes' reputational salience.

**Abbreviations**
GPA: grade point average
MCAR: missing complete at random
RCP: Revised Class Play
VIF: variance inflation factor

Child and early adolescent friendships are often unstable (e.g., Meter & Card, 2016). The proportion of Canadian elementary and middle‐school students' friendships that were maintained over a 3‐month interval ranged from 55% to 63% (Ellis & Zarbatany, 2007). More than 50% of mutual friendships of 4th‐ and 7th‐grade US children dissolved within a 3‐week period (Cairns et al., 1995) and approximately 37% of US sixth‐grade children's friendships formed in the fall semester ended by the spring semester (McDonald et al., 2013). Other friendships, however, are maintained for years.

Children with stable friendships experience intimacy and support and are more likely to influence each other (Berndt et al., 1986). In contrast, unstable friendships are associated with peer rejection and may indicate social maladaptation (Laursen, 2017); under some circumstances, terminating friendships with maladjusted peers reflects social competence (Flannery & Smith, 2017). In this study, we explored predictors of short‐term friendship stability of children in three countries. The participants were in the 5th grade, a period during which friendships are often unstable (Meter & Card, 2016).

Researchers (e.g., Hinde & Stevenson‐Hinde, 1976; Poulin & Chan, 2010) have suggested that child and adolescent friendship stability is a function of individuals' personal attributes and the characteristics of their relationship. The most comprehensive test of this hypothesis was provided by Hartl et al. (2015) who used discrete time survival analysis to assess the 5‐year stability of US 7th‐grade adolescents' friendships as a function of individual characteristic and dyadic similarity in peer acceptance, peer rejection, aggression, and school competence.

The prediction of short‐term friendship stability of US, Chinese, and Indonesian children from individual attributes and dyadic similarity in social preference, popularity, aggression, and academic achievement was examined in this study. Three of the variables (i.e., aggression, social preference, and academic achievement) were previously found to predict friendship stability in US and Western European children either as individual attributes (e.g., Dishion et al., 1995; Ellis & Zarbatany, 2007; Howes, 1990; Ng‐Knight et al., 2019) or dyadic similarity variables (e.g., Hartl et al., 2015).

Popularity was included as a predictor in this study because of its importance for US and Western European children's peer relationship and friendship selection (Cillessen et al., 2011; Dijkstra et al., 2013). Although popularity and social preference overlap, they differ conceptually and empirically. Whereas social preference reflects children's sentiments of liking others and integration within the peer group, popularity is characterized by reputational judgments of dominance, prestige, and visibility (Cillessen et al., 2011; van den Berg et al., 2020). A meta‐analysis revealed a moderate association between these two constructs (*r* = .45) (van den Berg et al., 2020), with correlations of similar magnitude found in China (Niu et al., 2016) and Indonesia (French et al., 2016). The correlates of popularity and social preference also differ. For example, aggression is positively associated with popularity but negatively associated with social preference in the United States (Cillessen et al., 2011), Indonesia (French et al., 2016), and China (Niu et al., 2016).

Chen and French (2008) expanded upon Hartup's (1996) concept of reputational salience to suggest that attributes that are valued within a culture are more strongly associated with individual adjustment than are others. The four variables selected as predictors in this study differ in reputational salience in the United States, China, and Indonesia and potentially account for country differences in the predictors of friendship stability. Based on evidence that will be discussed later, popularity and academic achievement appear to be particularly important respectively for friendships in the United States and China, and to a lesser extent there also country difference in the reputational salience of aggression and social preference. We explored the possibility that variation in reputational salience explains country differences in the predictors of friendship stability from individual characteristics and dyadic similarity.

This study was conducted in the United States, China, and Indonesia, three of the world's four most populous countries. The comparison of China and Indonesia is particularly interesting because although both cultures are considered collectivist, they differ in the importance of friendships. Whereas Chinese friendships tend to be very close, Indonesian friendships are of less importance and are lower in intimacy and exclusivity than friendships in either the United States or China (French, 2015).

## Individual characteristics and friendship stability

Hartup and Stevens (1997) suggested that friendship maintenance is influenced in part by individuals' interpersonal adjustment. Friendship may dissolve if one of the dyadic partners is aggressive or has poor social skills (Poulin & Chan, 2010). Conversely, the friendships of children who exhibit good social skills and other desirable qualities may last longer than those of others (Laursen, 2017). Although Hartl et al. (2015) did not find that individual characteristics predicted friendship stability, others have found opposite results.

Peer liking, peer disliking, or social preference (i.e., a combination of peer acceptance and rejection) predicted the stability of friendships. Friendships formed in kindergarten were almost nine times more likely to continue into the third grade for US children with higher levels of social preference than for others (Howes, 1990). Canadian kindergarteners who maintained their friendships over a 5‐month period were more socially preferred than were other children (Proulx & Poulin, 2013). As noted above, these findings stand in contrast to those of Hartl et al. (2015).

We were unable to find research pertaining to friendship stability as a function of children's popularity despite abundant research documenting the importance of this attribute for friendships (Cillessen et al., 2011). Ethnographic researchers (e.g., Eder, 1985; Merten, 1997) observed peer cliques comprised of popular US middle‐school students and reported that the memberships of the groups were stable and exclusive as popular children rejected the overtures of low‐status peers to join their group and chose each other as friends (Cillessen et al., 2011; Eder, 1985). We, therefore, anticipated that popular children would form stable friendships with those of similar or higher status.

Several researchers have found that friendships of aggressive and antisocial children are unstable (e.g., Dishion et al., 1995). Perhaps accounting for their difficulty maintaining friendships, antisocial youth exhibited low social skills, were negative and assertive with friends, and described their friendships as being low in quality (Dishion et al., 1995). Similarly, Canadian 5th‐grade children's overt aggression significantly predicted the dissolution of their friendships 3 months later regardless of the aggressiveness of their friends (Ellis & Zarbatany, 2007).

There are mixed results pertaining to the association between children's individual academic achievement and friendship stability. British primary‐school children's teacher‐rated academic attainment was positively associated with best friendship stability across the transition to secondary schools (Ng‐Knight et al., 2019). In contrast, school competence, a composite variable that included teacher‐rated academic performance, did not predict the friendship stability of 8th‐ to 12th‐grade US students (Hartl et al., 2015).

## Dyadic similarity and friendship stability

In addition to individual attributes, the similarity between friends also predicts friendship stability. The possibility that a mismatch between friends' attributes could threaten friendship stability has its roots in the extensive evidence that friends resemble each other on a variety of attributes including aggression, sociometric status, and academic achievement (e.g., Kupersmidt et al., 1995). This similarity, commonly labeled homophily, is a fundamental feature of friendships (Kandel, 1978) and may be partly a function of the low stability of friendships that exists when partners are dissimilar from each other. Based on social exchange theory, Laursen (2017) suggested that dissimilarity may result in conflicts and negative feelings that may lead to the terminations of friendships. Hartl et al.'s (2015) study of US adolescents' friendship stability from 7th to 12th grade provides the strongest evidence to date that similarity of friends predicts friendship stability: Dyadic similarity in peer acceptance, aggression, and school competence but not peer rejection predicted the survival of 7th‐grade friendships.

There is some evidence that similarity in popularity may predict friendship stability. Sixth‐grade students selected their best friends based on their similarity in popularity, an effect particularly strong for popular children (Dijkstra et al., 2013). Popularity similarity was more predictive of friendship selection of US 5th‐grade children than were prosocial behavior or aggression dyadic similarity (Logis et al., 2013). Because affiliation with low‐status peers can harm the status of US popular adolescents (Cillessen et al., 2011), they are likely to befriend others with status equal to or above their own (Dijkstra et al., 2013). Although popularity similarity has been shown to impact friendship selection, there are no studies to date exploring the effects of this on friendship stability.

Several researchers have found that dyadic similarity in aggression and antisocial behavior predicts friendship stability. The friendships of Dutch 12‐year‐old adolescents were more stable for partners similar in aggression over a 7‐month period than for those that differed (Laninga‐Wijnen et al., 2017). Dyadic similarity in other problem behaviors, such as truancy (Rambaran et al., 2017) or substance use (DeLay et al., 2013), also positively predicted friendship stability.

Consistent with the findings of Hartl et al. (2015), friendships were more stable when partners are similar in academic achievement. US 9th‐grade students formed and maintained their friendships with peers with similar levels of Grade point average (GPA) over a two‐year period, an effect that was stronger for low‐achieving friend partners than for high‐achieving dyads (Rambaran et al., 2017). In addition, US middle‐ and high‐school students were more similar to their stable friends in academic motivation than they were with either nonfriends or unstable friends (Hafen et al., 2011).

## Culture and friendship stability

Friendships exist within a cultural context in which meaning systems are pertinent to understanding the relative importance of friendship and provisions that individuals expect to receive from these relationships (Chen & French, 2008; Oh et al., 2021). Surprisingly, we could find no research assessing friendship stability across cultures despite the importance of this topic (Rubin et al., 2011). We addressed this lacune by exploring friendship stability in the United States, China, and Indonesia with the hypothesis that cultural norms pertaining to friendship and the desired attributes of these relationships are pertinent to understanding the predictors of friendship stability.

The relative importance of behavioral characteristics across countries has been conceptualized using the “reputational salience” framework developed by Hartup (1996). Chen and French (2008) expanded upon Hartup's (1996) argument by suggesting that cultural differences in the extent that attributes are emphasized or pertinent to cultural norms are likely to have a greater influence on children's outcomes than are others. We explored the possibility that individual and dyadic similarity attributes of social preference, popularity, aggression, and academic achievement explain friendship stability across the United States, China, and Indonesia in a way that is consistent with our knowledge of the reputational salience of these attributes in these countries.

We hypothesized that social preference as both an individual and dyadic similarity attribute will be associated with friendship stability across countries, but these associations may be stronger for Chinese and Indonesian than for US children. Zhang et al (2024), in an analysis using some of the current data, revealed that after controlling for popularity, social preference negatively predicted loneliness for Chinese but not US children. The authors interpreted these findings by suggesting that connectedness with others and group integration as indicated by social preference are more strongly valued for Chinese children than for their US counterparts. Similarly, the maintenance of harmonious interpersonal relationships is also endorsed in Indonesia (French et al., 2012).

Although popularity is important for US, Chinese, and Indonesian children's peer relationships (Cillessen et al., 2011; French et al., 2016; Niu et al., 2016), popularity may be more reputationally salient for US than for Chinese children. This hypothesis is in part based on Zhang et al. (2024) findings that popularity predicted reduced loneliness for US but not for Chinese 5th‐grade students. Additional evidence that popularity is also less salient for Chinese children's friendship than for that of US children comes from Li et al.'s (2012) finding that Chinese 5th‐grade children were significantly less likely to attribute social connections (i.e., having a lot of friends and hanging out with popular students) as a determinant of popularity than did their US counterparts. The importance of the individual attribute or dyadic similarity in popularity for Indonesian children's friendship stability is unknown.

Aggression is expected to negatively predict friendship stability across countries, an effect that may be stronger for Chinese and Indonesian than for US children. A meta‐analysis revealed that aggression was more strongly associated with peer rejection in collectivistic than in individualistic cultures (Yue & Zhang, 2023). Consistent with the finding of this meta‐analysis, the negative association between aggression and social preference in the 5th grade was greater for Chinese students than for US children (Zhang et al., 2024) . Aggression is also less tolerated in Indonesia than in the United States. Indonesian elementary school children more frequently mentioned physical aggression than did their US counterparts when describing disliked peers (French et al., 2002). Because of the negative view of aggression, we expected that aggressive children would have difficulty sustaining friendships across countries, particularly in China and Indonesia. There is evidence from the United States that aggressive and antisocial children tend to form relationships with each other, but their friendships dissolved quickly (Dishion et al., 1995) and we expected that this would be particularly the case in China and Indonesia where aggression is strongly discouraged.

Finally, individual and dyadic similarity in academic achievement may be a stronger predictor of friendship stability for Chinese than for US children. Parents, teachers, and peers in Asian cultures are strongly influenced by Confucianism to value academic success (Li et al., 2012). Chinese middle‐ and high‐school students selected friends based on their similarity in academic performance, an effect greater for academically successful friend dyads than for low‐achieving dyads (Shen & French, 2024). Academic achievement similarity was also expected to predict Indonesian children's friendship stability, although perhaps not as strongly as it does for Chinese children.

## Cross‐classified multilevel modeling to analyze friendship stability

Two challenges are pertinent to analyzing friendship stability. First, children often have multiple friendships, and the stability of their friendships are interdependent. For example, three unique friendships can emerge between Child A, B, and C (i.e., A and B, A and C, B and C). Because a child can be either a focal child or a friend of the focal child, each friendship needs to be considered twice, resulting in six dyad combinations (i.e., A‐B and B‐A, A‐C and C‐A, B‐C and C‐B) (Kenny et al., 2020). Because Child A is the focal child in Dyad A‐B and Dyad A‐C, these two friendships are interdependent because Child A's individual attributes may influence the stability of both. In addition, friendship stability is influenced by characteristics of both partners. For example, Child A and B are, respectively, the focal children in Dyad A‐C and Dyad B‐C and Child C is their common friend. The stabilities of Dyad A‐C and Dyad B‐C are not only associated with the individual attributes of Child A and Child B but also likely influenced by those of Child C.

Cross‐classified multilevel analyses were used to address the two challenges noted above (Fielding & Goldstein, 2006). First, this analysis employs a multilevel structure that allows children to have multiple friendships and consequently accounts for stability of multiple friendships that arise from the shared influence of children's individual attributes. Second, this analysis allows friendships to be classified within both focal children and their friends and thus includes both partners' attributes. For example, Child A and Child B's unique influence on the stability of Dyad A‐C and Dyad B‐C can be assessed with Child C's attributes also included in the model. More examples and detailed introduction of this analysis are provided in Supporting Information.

## The current study

Fifth‐grade children from the United States, China, and Indonesia were followed from the fall into the spring semester. Students nominated their classroom friends at each of two timepoints and stable and dissolved reciprocated friendships were identified. There were three major aims.

The first aim was to assess the main effects of individual attributes on friendship stability. Based on the arguments presented earlier, individual attributes of social preference, popularity, and academic achievement were expected to positively predict friendship stability, whereas aggression was expected to predict low friendship stability. It is also possible, however, that individual attributes will not predict friendship stability given the results from Hartl et al. (2015). The tests of main effects for individual attributes were confirmatory.

The second aim was to examine the effects of dyadic similarity on friendship stability. Friendships in which dyads were similar in social preference, popularity, aggression, and academic achievement were expected to be more stable than those in which there was a mismatch; these assessments were confirmatory.

Secondary to the first and second aims were exploratory analyses of sex moderation of individual and dyadic similarity predictors of friendship stability We located only three published quantitative studies pertinent to the hypothesis. Hartl et al. (2015) found that sex did not moderate the prediction of friendship stability from either individual attributes or dyadic similarity. Using the same date set, Guimond et al. (2019) found that submissiveness similarity was more predictive of girls' friendship stability than that of boys, but no sex moderations emerged for similarity in depression and anxiety or for individual internalizing problems. Canadian 8th‐grade adolescents' individual aggression and social preference, respectively, predicted lower and higher friendship stability for boys and girls, although the association between friendship stability and dyadic similarity in victimization was stronger for girls than for boys (Ellis & Zarbatany, 2007). Despite the limited evidence of sex moderation of predictors of friendship stability, we analyzed this for two reasons. First, the quantitative evidence pertaining to this issue is thin, consisting of three studies with mixed findings. More important is prior evidence of sex differences in children's peer relationships (Rose & Smith, 2018) and the maintenance of friendships (Azmitia et al., 1997).

The final aim was to conduct exploratory analyses assessing the possibility that country moderated the prediction of friendship short‐term stability from individual and dyadic similarity attributes. We speculated that social preference, aggression, and academic achievement would more strongly predict friendship stability for Chinese than for US children. In contrast, we expected that the effects of popularity for individual and dyadic similarity would be stronger for US than for Chinese children. Because of limited information comparing the reputational salience of attributes of Indonesian children with that of either US or Chinese children, we are reluctant to make strong predictions pertaining to the predictors of friendship stability for these children but expected that these would be more similar to Chinese than to US children.

## METHOD

### Participants

Fifth‐grade US, Chinese, and Indonesian students were assessed two times within one academic year (labeled T1 and T2). Initial US, Chinese, and Indonesian samples, respectively, included 540, 576, and 489 children. Assessments were administered in the National languages in each country (i.e., English, Mandarin, and Bahasa Indonesia).

Data from children that participated at both time points were used in the analyses to avoid confounding attrition with friendship dissolution (Nielson et al., 2020), resulting in a sample of 510 US, 569 Chinese, and 462 Indonesian children. Attrition analyses revealed no differences between children that participated at both T1 and T2 with those that completed only the T1 assessment. Children without friends at T1 were also omitted from the analysis, resulting in 477 US (238 girls; *M*_age_ = 10.2 years, SD = .41), 467 Chinese (275 girls; *M*_age_ = 10.4 years, SD = .54), and 419 Indonesian (190 girls, *M*_age_ = 10.3 years, SD = .54) children. US, Chinese, and Indonesian children without a mutual friend at T1 (respective *n* = 33, 102, and 43) were less socially preferred than were those with one or more friends (respective *d* = −1.32, −.52, and −1.01). Compared to children that had at least one friend, friendless US and Indonesian children at T1 were less popular (respective *d* = −1.05 and −.34) and US and Chinese children were more aggressive at T1 (respective *d* = .61 and .53). Chinese children without friends at T1 had lower academic achievement than did those with friends (*d* = −.33).

Data for US children came from a study of classroom peer ecologies conducted in Illinois, Indiana, and Pennsylvania (*reference blinded for review*). The US data collection occurred from 2009 to 2012; T1 data were obtained in December and T2 data were obtained in May within the same academic year. Small rural schools were excluded to enhance the comparability between the US and the Chinese and Indonesian urban schools. Participants came from 31 classrooms in 14 community schools. The sample included 40% African American, 35% White, 14% Latino, 6% Asian, and 5% other. The highest education levels within each family for the US samples were 5.4% with an education below high school; 14.3% with a high‐school diploma; and 80.3% with some college education. More than half of the participants (59.7%) were enrolled in the “Free/Reduced Price Lunch” program, indicating the low‐income status of many participants.

Chinese participants were recruited from 13 classrooms in Shanghai public schools. The T1 Chinese data was obtained during October or November 2013 and the T2 data were obtained in June or July 2014. The highest parental education levels with each family were 7% with an education below high school; 27.5% with a high‐school degree; and 65.6% with a college degree. Monthly family income was: 21.9% (less than $1000); 53.6% ($1000 to $3000); and 24.5% (more than $3000). Almost all Chinese were of Han ethnicity.

Indonesian students came from 15 classes in three private elementary schools located in Bandung, Java. The T1 data collection occurred during September and October 2013 and the T2 data were obtained during April and June 2014. The highest family education was 2.8% with an education below high school; 31.8% with a high‐school or technical‐school education; 66.2% with a college or post‐college degree. Almost all students (98.8%) were Muslim, and 76% were Sudanese, 14% were Javanese, and the remainder were members of other Indonesian ethnic groups.

Chinese children were older than US children (*d* = .41) and Indonesia children (*d* = .24). There was no country difference in the percentage of boys and girls across countries (*χ* ^2^(1) = .64, *p* = .43). No country differences were found for the proportion of parents with a college degree (*χ* ^2^(2) = 2.89, *p* = .23). Parent education was unrelated to friendship stability in any country (*r* = .05, .01, and −.05, respectively, for the United States, China, and Indonesia, *p*s > .05).

### Measures

#### Friendships

Children nominated an unlimited number of friends of either sex by circling names on class lists. At T1, there were 2643 unique same‐sex reciprocated friendships (US *n* = 1022, 50% girl dyads; China *n* = 975, 55% girl dyads; Indonesia *n* = 646, 39% girl dyads). There were 104 cross‐sex dyads (*n* = 68, 17, and 19, respectively, for the United States, China, and Indonesia). Because cross‐sex friendships were infrequent and likely differed from same‐sex friendships (Lempers & Clark‐Lempers, 1993), these were not included in the analyses. Friendship stability was assessed by examining whether reciprocated friendships at T1 were also present at T2. Friendships were classified as unstable if either of the partners did not nominate the other at T2 (0 = instable, 1 = stable) (Nielson et al., 2020).

#### Peer nominations

Social preference, popularity, and aggression were (RCP; Masten et al., 1985). For each RCP item, children circled the names of classmates of either sex using unlimited nominations. The RCP has been extensively used in the United States, China, and Indonesia with evidence of good reliability and construct validity (e.g., Chen et al., 1995; French et al., 2016). The total nominations for each item were standardized within classrooms using *z*‐scores.

Social preference was computed from single items that assessed peer acceptance and peer rejection (i.e., “These classmates you like the most” and “These classmates you like the least”). Social preference was computed by subtracting the standardized score of peer acceptance from the standardized score of peer rejection. Popularity was assessed by one item (i.e. “These classmates are popular”). Aggression was the mean scores of three items (i.e., “These classmates like fighting, pushing, beating, or kicking others”, “These classmates say mean things”, and “These classmates tease others”) that were standardized within classrooms. The internal consistency of aggression was between .91 and .93.

#### Academic achievement

Fall‐semester‐end math and language exam scores were obtained from school records, The mean of two subjects was computed and standardized within classrooms using *z*‐scores. Respective correlations between these two subjects were .70, .55, and .71 for US, Chinese, and Indonesian children.

### Analysis

Four dyadic similarity variables were created following the procedures outlined by Nielson et al. (2020). First, difference scores between friends for the four variables measured at T1 (i.e., social preference, popularity, aggression, and academic achievement) were computed. The absolute values of the scores were computed and reversed such that higher values indicated greater similarity between friends. Individual and dyadic similarity scores were standardized within each country.

Cross‐classified multilevel regression analyses were used to predict friendship stability from individual attributes and dyadic similarity using the *lme4* package in *R*. Dyadic similarity variables were entered as Level 1 friendship‐level predictors, and individual attributes were included as Level 2 child‐level predictors. The number of friendships of both focal children and their friends were computed and entered as controls (Nielson et al., 2020). Sex, country, and friends' attributes were also included as controls. Because of multicollinearity concerns variance inflation factor (VIFs = 1.05 to 2.42; Kim, 2019), four independent models for social preference, popularity, aggression, and academic achievement were constructed. Four two‐way interactions (i.e., sex by individual attribute, sex by dyadic similarity, country by individual attribute, and country by dyadic similarity) were entered to assess sex and country moderation effects. Non‐significant interactions were excluded from the final models. In the first analysis, Chinese students were the reference group and were compared to US and Indonesian children. Then, US children were selected to be the reference and compared with Indonesian children. Equations for each step of assessment are included in Supporting Information.

There were no missing values for social preference, aggression, or popularity. Following the steps outlined by Jakobsen et al. (2017), the percentage of missing data for academic achievement in the three samples was computed. Because this was negligible for China (1.3%) and Indonesia (2.4%) but not for the United States (11.5%), Little's missing complete at random (MCAR) test was applied to the US data with the result that there was a MCAR pattern (*χ* ^2^(5) = 7.85, *p* = .17). Multiple imputation was used to adjust for missing data.

### Procedure

Across samples, parents and students respectively provided written consent and assent. Data from six US classrooms were omitted because the parental consent rates of these classrooms were below 70%; this resulted in 85% approval. The US research was overseen by Pennsylvania State University and the University of Illinois IRBs. The Chinese participation rate was 95%, and was overseen by the Shanghai Normal IRB . The Indonesian participation rate was 96%, and this research was overseen by the Purdue University IRB.

## RESULTS

### Descriptive analyses

Table 1 provides descriptive information of friendships, individual attributes, dyadic similarity variables for children with complete participation that had one or more reciprocated T1 friendships. Also included in this table are the ANOVA results that assessed sex and country differences and sex by country interactions.

**TABLE 1:** Means, standard deviations of friendship‐level and child‐level variables and differences across sex and cultures.

	US		China		Indonesia		*F*‐value		
	Girls	Boys	Girls	Boys	Girls	Boys	Country	Sex	C × S
Friendship attributes									
Popularity similarity	0.11 (0.89)	−0.11 (1.09)	0.15 (1.09)	−0.17 (0.86)	0.16 (0.88)	−0.07 (1.06)	0.61	41.06***	0.63
Preference similarity	0.01 (0.99)	−0.01 (1.01)	0.04 (0.93)	−0.04 (1.07)	0.21 (0.88)	−0.14 (1.05)	0.39	14.09***	5.60**
Aggression similarity	0.08 (0.91)	−0.08 (1.08)	0.28 (0.62)	−0.32 (1.24)	0.47 (0.41)	−0.30 (1.14)	2.43	167.77***	23.05***
Academic similarity	0.01 (1.01)	−0.01 (0.99)	00.00 (1.03)	.00 (0.96)	0.14 (0.89)	−0.09 (1.06)	0.11	3.94	2.74
Friendship stability	0.67 (0.47)	0.73 (0.45)	0.58 (0.49)	0.52 (0.50)	0.53 (0.50)	0.49 (0.50)	37.04***	0.79	3.78*
Individual attributes									
Popularity	−0.12 (0.97)	−0.11 (1.08)	−0.12 (0.95)	−0.19 (0.81)	−0.18 (0.84)	−0.01 (1.02)	0.48	0.55	1.77
Preference	−0.27 (1.94)	−0.54 (2.01)	−0.16 (1.71)	−0.82 (1.97)	−0.26 (1.59)	−0.25 (1.90)	1.81	9.19**	3.59*
Aggression	−0.08 (0.92)	0.22 (1.18)	−0.16 (0.74)	0.36 (1.25)	−0.47 (0.43)	0.28 (1.16)	2.84	91.61***	5.57**
Academic	−0.11 (1.01)	−0.03 (1.89)	0.07 (1.01)	−0.26 (1.06)	0.04 (0.93)	−0.04 (1.04)	0.95	3.87*	4.34**
Number of friends	4.31 (2.05)	4.26 (1.89)	4.41 (2.59)	3.93 (2.88)	2.64 (1.61)	3.45 (1.99)	41.11***	0.57	9.49***

*Note*: Standard deviations are in the paratheses. Friendship‐level samples include 2463 unique friend dyads (1022. 975, and 646, respectively for the US, Chinese, and Indonesian sample). Individual‐level samples include 1363 children (477, 467, and 419 respectively for the US, Chinese, and Indonesian sample).

* *p* < .05;

** *p* < .01;

*** *p* < .001.

The main effect of country on the number of friendships was moderated by sex. Indonesian girls had fewer friends than did Chinese or US girls (respective *d* = −.91 and −.82), whereas Chinese and US girls did not differ. Indonesian boys had fewer friends than did US boys (*d* = −.42); Chinese boys did not differ from either Indonesian or US boys.

There was a main effect of country and a country by sex interaction for friendship stability. The proportion of stable friendships (see Table 1 for percentages of stable friendships by country and sex) was higher for US than for Chinese and Indonesia children and the proportion of stable friendships for US boys was greater than that of Chinese and Indonesian boys (respective *d* = .44 and .50); US girls also had more stable friendships than Chinese and Indonesian girls (respective *d* = .19 and .29). The country differences for boys were significantly larger than those for girls.

#### Friendship similarity: Sex and country differences

Indonesian girls were more similar in social preference to their friends than were boys (*d* = .36), a difference that was not significant for US and Chinese children. Across the three countries, girls were more similar to their friends in popularity than were boys (*d* = .23). The difference between boys and girls for aggression dyadic similarity was also moderated by country; girls were more similar to their friends than were boys. This effect was larger for Chinese and Indonesian children (respective *d* = .62 and .82) than for US children (*d* = .15). No sex, country, nor sex by country interaction effects were identified for academic achievement dyadic similarity.

#### Individual attributes: Sex and country differences

No sex, country, or sex by country interaction effects were found for popularity. The sex difference in social preference was moderated by country; girls were more socially preferred than boys in China (*d* = .36), whereas this sex difference was not significant for US or Indonesian children. Across countries, boys were more aggressive than girls, an effect that was larger for Chinese (*d* = −.51), and Indonesian (*d* = −.83), than for US children (*d* = −.28). The academic achievement sex effect was also moderated by country; Chinese girls had higher academic scores than boys (*d* = .32), a sex difference not found for either US or Indonesian children.

#### Correlations

Zero‐order point‐biserial correlations between friendship stability and predictors are included in Table 2. Friendship stability was positively associated with individual popularity and social preference across countries. Individual academic achievement and friendship stability emerged for Chinese and US but not for Indonesian children whereas aggression was negatively associated with friendship stability in China and Indonesia. Dyadic similarity in social preference was positively associated with friendship stability for children in all countries. Aggression similarity was positively correlated with friendship stability only for Indonesian children and academic achievement similarity was positively associated with friendship stability only for Chinese children. Associations between all variables for US, Chinese, and Indonesian children are provided in Tables S1–S3.

**TABLE 2:** Zero‐order point‐biserial correlations between friendship stability and dyadic and individual attributes in the US, Chinese, and Indonesian sample.

	US	China	Indonesia
Popularity similarity	−.01	.04	−.02
Preference similarity	.06**	.06**	.11**
Aggression similarity	.03	.03	.14**
Academic similarity	−.02	.11**	.04
Individual/friend popularity	.14**	.07**	.05*
Individual/friend preference	.08**	.05*	.13**
Individual/friend aggression	.02	−.04*	−.08**
Individual/friend academic	.06**	.09**	.04
Individual/friend number of friends	.10**	−.07**	−.01

*Note*: Samples included 5286 double‐entered friendship dyads (2044, 1950, and 1292 respectively for the US, Chinese, and Indonesian sample). Friendship stability was identically associated with child individual attributes and friends' attributes because children are both focal children and friends (e.g., A's contribution to A‐B in which A is the focal child = A's contribution to B‐A in which A is the friend).

* *p* < .05;

** *p* < .01.

### Preliminary model testing

Table S4 presents model fit indices, and the results from likelihood ratio tests that assessed model improvement resulting from the inclusion of covariates, individual attributes, dyadic similarity variables, and significant interactions. An intraclass correlation coefficient was computed based on the unconditional baseline model with the result that approximately 16.8% of the variance of children's friendship stability was attributable to differences between children. Because none of the sex by individual attribute, sex by dyadic similarity variable, or country by individual attribute interactions were significant, these interactions were not included in the models.

### Individual attributes, dyadic similarity variables, and country moderations

Table 3 presents the results of the four cross‐classified multilevel models for social preference, popularity, aggression, and academic achievement. Discussed below are the main effects for individual attributes, dyadic similarity, and the interactions of these with country. No sex moderation emerged for any individual or dyadic similarity predictors.

**TABLE 3:** Fixed effects and standard errors for models predicting friendship stability.

	Popularity		Social preference		Aggression		Academic achievement	
	*b*	SE	*b*	SE	*b*	SE	*b*	SE
Main effects								
Level 1: friendship‐level								
Dyadic similarity	.001	.007	.023**	.007	.036***	.010	.021**	.007
Level 2: child‐level								
Individual/friend attribute	.036***	.009	.019***	.005	.002	.010	.026**	.009
Sex (0 = girl, 1 = boy)	−.003	.019	.013	.019	.012	.020	.005	.010
Country: US versus China	.116***	.022	.116***	.022	.120***	.023	.120***	.023
Country: Indonesia versus China	−.060*	.025	−.070**	.025	−.053*	.035	−.056*	.025
Country: Indonesia versus US	−.174***	.024	−.185***	.024	−.173***	.025	−.175***	.024
Individual/friend number of friends	−.002	.004	−.004	.004	.001	.004	.000	.004
Significant two‐way interactions								
Similarity × Country (US vs. China)	.048**	.017	‐	‐	‐	‐	−.043**	.016
Similarity × Country (Indonesia vs. China)	‐	‐	‐	‐	.071***	.020	‐	‐
Similarity × Country (Indonesia vs. US)	‐	‐	‐	‐	.062**	.019	‐	‐

*Note*: Samples included 5286 double‐entered friendship dyads (2044, 1950, and 1292 respectively for the US, Chinese, and Indonesian sample). Estimations for individual and friend attributes are identical because friendships were doubly entered and children's contributions to their own friendships were the same as their contributions to friendships in which they acted as friends.

Abbreviation: SE, standard errors.

* *p* < .05;

** *p* < .01;

*** *p* < .001.

#### Social preference

Friendship stability was positively predicted by both individual social preference (95% CI [.010, .029]) and dyadic social preference similarity (95% CI [.009, .037]). No significant country moderations emerged.

#### Popularity

Individual popularity predicted friendship stability (95% CI [.019, .053]), an effect that was not moderated by country. The main effect for popularity dyadic similarity was not significant, but the significant interaction with country revealed that this was significant for US but not for Chinese or Indonesian children. This effect was significantly stronger for US than for Chinese children (95% CI [.016, .081]); the difference between US and Indonesian children and the difference between Chinese and Indonesian children were not significant.

#### Aggression

Children's friendship stability was not significantly associated with individual aggression, an effect that did not differ across countries. Friendship stability was positively predicted by aggression dyadic similarity (95% CI [.016, .054]), but the significant aggression dyadic similarity by country interaction revealed that the effect was present for Indonesian and US, but not for Chinese children. The strength of the association was significantly greater for Indonesian than for US (95% CI [.024, .098]) and Chinese children (95% CI [.032, .109]); Chinese and US children did not differ.

#### Academic achievement

Individual academic achievement positively predicted friendship stability (95% CI [.009, .042]), an effect that was not moderated by country. The main effect of academic achievement dyadic similarity was positive and significant (95% CI [.007, .034]), but the significant country moderation effect revealed that Chinese but not US or Indonesian children's friendship stability was positively predicted by academic achievement dyadic similarity. This association was significantly greater for Chinese than for US children (95% CI [−.075, −.011]). Indonesian children did not significantly differ from either Chinese or US children.

## DISCUSSION

This study was conducted to explore the prediction of short‐term friendship stability from individual and dyadic similarity attributes for US, Chinese, and Indonesian children. The first and second aims were to identify the extent that individual or dyadic similarity attributes explained friendship stability. The third aim was to examine country moderation effects on the associations between individual and dyadic similarity attributes and friendship stability. There was evidence that both individual and dyadic similarity attributes predicted friendship stability although the predictions from dyadic similarity attributes varied by country in ways that were consistent with the reputational salience of the attributes.

### Individual attributes and friendship stability

There were significant main effects that positively predicted friendship stability from individual attributes of social preference, popularity, and academic achievement. These effects were not moderated by sex. These results are consistent with the argument that friendships are more sustained by children with desirable qualities (Hartup & Stevens, 1997; Howes, 1990).

Although individual aggression was expected to predict friendship stability, the results did not support this hypothesis. Although negative and small zero‐order associations between individual aggression and friendship stability emerged for both Chinese and Indonesian children (respective *r* = −.04 and −.08), these effects were not significant in the comprehensive model that included both individual and dyadic similarity in aggression. Perhaps the principle effects of individual aggression are a failure to form friendships rather than the instability of friendships that were formed.

The finding that most individual attributes were associated with friendship stability are inconsistent with the findings of Hartl et al. (2015). There are many differences between the studies that could account for the disparity in the results. One possibility is that Hartl et al. (2015) followed US 7th‐grade students' friendships to the 12th grade whereas in the present study, friendship stability was assessed for 5th‐grade children during one academic year. Perhaps individual characteristics may be particularly important for short‐term friendship stability.

### Dyadic similarity and friendship stability

The analyses yielded main effects predicting friendship stability from dyadic similarity in social preference, aggression, and academic achievement but not popularity. No sex differences emerged for these effects but some of these differed by country in ways explainable by the reputational salience of attributes.

The finding that friendships were more stable for partners similar to each other is consistent with other findings. Dutch 12‐year‐old students' friendship maintenance was a function of aggression similarity between friends (Laninga‐Wijnen et al., 2017) and US ninth‐grade adolescents retained friendships with those with similar levels of GPA (Rambaran et al., 2017). Dyadic similarity in aggression, peer acceptance, and school competence predicted friendship survival of US 7th‐grade students (Hartl et al., 2015). Although there was no main effect for popularity similarity, the effect was significant for US children. As we discuss below, this result is consistent with our hypothesis that popularity is more reputationally salient for US than for Chinese or Indonesian children.

### Sex moderation of the prediction of friendship stability

Our findings were consistent with Hartl et al.'s (2015) findings that sex did not moderate the prediction of friendship stability from either individual attributes or dyadic similarity. There are several reasons, however, why researchers should continue to explore sex differences and friendship stability. First, Hartl et al. (2015) and we assessed friendship stability respectively over a one year or one semester time span. Friendships, however, change rapidly during childhood and sex differences in friend stability may only emerge when this is assessed over a shorter period (Cairns et al., 1995). Second, boys and girls may substantially differ in the processes by which they retain friendships, differences not reflected in the duration of friendships (Meter & Card, 2016) nor in the predictors of friendship stability (Hartl et al., 2015). Two qualitative studies (Azmitia et al., 1997; Benenson & Christakos, 2003) suggested that girls are more sensitive to the violations of friendships norms than boys and differ in the processes they use to address these. Although girls and boys are similar in the length of friendships (Meter & Card, 2016), the extent to which the strategies used for sustaining friendships differ across sex requires further exploration.

Gender schema theory suggests that children apply cultural rules pertaining to appropriate sex role behavior (Bem, 1983), and it may prove worthwhile to study these across cultures. Gender schema theory may be a useful complement to the reputational salience model by suggesting that this is not only a function of culture but also gender. Thus, in addition to studying the behavior of boys and girls across cultures, it may prove useful to explore children's and adult's perspectives pertaining to sex roles in friendships and expectations of behavior within these relationships with the view that these beliefs are pertinent to friendship stability.

### Culture and friendship stability

Based on the reputational salience framework (Chen & French, 2008; Hartup, 1996), friendship stability was expected to be associated with individual and dyadic similarity attributes to the extent that these are particularly valued within the culture. Contrary to our expectations, the effects of individual predictors did not differ by country. Based on past research, social preference, popularity, academic achievement, and low aggression are associated with social competence across cultures (Chen & French, 2008; Flannery & Smith, 2017) and perhaps the contribution of personal qualities to friendship stability also generalizes across countries. Given that this is the first study to explore culture and friendship stability, further comparisons across multiple countries are needed.

Country moderated the associations of friendship stability with three of the four dyadic similarity variables, the exception being social preference. Sustaining friendships is a decision‐making process whereby children evaluate the gains and loss associated with friendship maintenance (Laursen, 2017) and they may emphasize similarity with friends on attributes considered important in their culture.

#### Social preference

Although we hypothesized that the positive association between social preference and friendship stability similarity would be stronger for Chinese and Indonesian children than for their US counterparts, the results revealed that this association did not differ across countries. Perhaps social preference is important for friendship stability regardless of culture. It may be useful in future studies to conduct further analyses that unpackage the social preference construct (Asher & Coie, 1990).

#### Popularity

Popularity similarity was significantly and positively associated with friendship stability for US but not for Chinese and Indonesian children; the differences between the United States and Indonesia and between China and Indonesia were not significant. The significant country difference between the United States and China is consistent with the expectation that popularity is more reputationally salient for US children than for their Chinese counterparts (e.g., *reference blinded for review*). Popularity similarity did not predict Indonesian children's friendship stability, suggesting that popularity in Indonesia may not be as salient as it is in the United States, although the behavioral profiles of popular adolescents are similar in Indonesia and United States (French et al., 2016). Perhaps the visibility and social dominance associated with popularity (Cillessen et al., 2011) are less valued in countries such as China and Indonesia in which it is particularly important for children to fit into the group (Ho, 1986). Unlike US children that may end their friendships with non‐popular peers to avoid losing status (Eder, 1985), Chinese and Indonesian children may be more accepting of others that differ from them in popularity.

#### Aggression

The positive association between aggression dyadic similarity and friendship stability was present in Indonesia and the United States but not in China. This effect was significantly stronger for Indonesian than for Chinese or US children; Chinese and US children did not significantly differ. The reputational salience of aggression in Indonesia may stem from the cultural emphasis on maintaining overtly harmonious social relationships. Exhibiting overt confrontation and direct coercion violates the “*Rukun*” principle that values harmony in face‐to‐face interactions (Magnis‐Suseno, 1997). Indonesian children, therefore, may terminate their friendships with aggressive peers. Aggressive children, however, may be able to maintain friendships with other aggressive peers partly because their other friendship choices are limited.

The hypothesized positive association between aggression similarity and friendship stability for Chinese children did not emerge. Perhaps this is a function of the lack of differentiation between subtypes of aggression in this study (e.g., physical vs. verbal, proactive vs. reactive). French et al.'s (2022) finding that aggression coupled with high levels of effortful control positively predicted Chinese middle‐ and high‐school youth leadership suggests the possibility that aggression is tolerated in China when it is used skillfully with high self‐control. Future research may explore if aggression (either as an individual attribute or a dyadic similarity variable) predicts Chinese children's friendship stability as a function of different types of aggression.

#### Academic achievement

Academic achievement similarity positively predicted friendship stability of Chinese but not children in the United States or Indonesia. Significant country differences emerged only between the United States and China. The finding that academic achievement similarity predicted Chinese but not US children's friendship stability is consistent with the expectation that academic success is more reputationally salient in China than it is in the United States. The reputational salience of academic success for Chinese children is also supported by evidence that academic engagement predicted increased popularity in the subsequent semesters for Chinese but not for US 7th‐grade students (Zhang et al., 2018). Shen and French (2024) reported that academically successful Chinese middle‐ and high‐school youth more often developed friendships based on similarity in academic achievement than did low‐achieving students and concluded that students sought to develop friendships with higher‐achieving peers. Chinese middle‐school children also attributed academic competence as a more important determinant of popularity than did their US counterparts (Li et al., 2012).

The finding that academic achievement similarity did not predict the stability of US children's friendships is inconsistent with Hartl et al.'s (2015) finding that teacher‐rated school competence predicted the maintenance of US 7th‐grade students' friendships over 5 years (Hartl et al., 2015). Two potential differences between the present study and that of Hartl et al. (2015) may contribute to this discrepancy. First, academic achievement in the present study was obtained from school records rather than from teacher reports that may be influenced by qualities in addition to academic achievement. Second, Hartl et al. (2015) assessed adolescents' friendship stability over many years.

It was anticipated that the effect of academic achievement similarity on Indonesian children's friendship stability would be significant but weaker than that for Chinese children. Although the results were consistent with this hypothesis, the country difference was not significant. French et al.'s (2016) finding that academic success did not predict Indonesian middle‐ and high‐school adolescents' popularity, suggests the possibility that academic success may not be highly reputationally salient in this population.

### Limitations and direction for future research

This is one of few studies to directly compare individual and dyadic similarity attributes as predictors of friendship stability across countries. A major strength of this study is the comparison of four attributes that predicted children's friendship stability in three countries. An additional strength includes the use of cross‐classified multilevel analysis that included individual and dyadic similarity attributes predictors in a single analysis. Several limitations, however, exist that suggest the need for future study.

First, there are other individual attributes or dyadic similarity qualities potentially important for friendship stability that were not assessed in the current study. For example, individual victimization was negatively associated with friendship stability of Canadian children (Ellis & Zarbatany, 2007). Dyadic similarity in depression, anxiety, and submissiveness positively predicted middle‐school adolescents' friendship stability (e.g., Guimond et al., 2019). Dyadic similarity in victimization (Ellis & Zarbatany, 2007) and happiness (van Workum et al., 2013) also positively predicted the friendship stability respectively of Canadian 8th‐grade girls and US middle‐school students.

Second, we did not assess the quality of children's friendship. Berndt et al. (1986) found that US 4th‐ and 8th‐grade students reported positive experiences (e.g., high intimacy, loyalty or faithfulness, and similarity) with their stable friends whereas they reported negative experiences with unstable friendship partners. Including assessment of children's perceived friendship quality could clarify the reasons why some individual characteristics and dyadic similarity are associated with friendship stability. For example, Dishion et al. (1995) found that the instability of antisocial boys' friendships was attributable to their negative perceptions of these relationships. Laursen (2017) argued that dissimilarity predicts escalated conflicts between partners that account for friendship termination.

Third, we were unable to identify the length of each friendship because the lag between the first and second assessment was a maximum of 8 months and consequently it is unknown when these friendships began or when they ended. It will be important for researchers to assess friendships over multiple time points that span multiple years (e.g., Guimond et al., 2019; Hartl et al., 2015). Additionally, the spacing between the two points of friendship assessments in the present study varied across countries (approximately 6 months for the United States and 7 to 8 months for the Chinese and Indonesian samples). It will be important researchers to address this possible confound given findings that time between assessments is negatively associated with friendship stability (Meter & Card, 2016).

Fourth, in additional to differences in cultural norms, the United States, China, and Indonesia differ substantially in economic prosperity as evidenced by their rankings in the United Nations Human Development Index 2023–2024. Of 189 countries, these were respectively 20, 75, and 112. The wide income and educational disparities between these countries is undoubtably reflected in the samples. Unfortunately, we were unable to control this potential confound, and it remains a task for future researchers to continue to explore how SES and culture are intertwined. Initial efforts to explore how cultural values are associated with economic prosperity is provided by the World Values Survey (Haerpfer et al., 2022) and Lansford et al.'s (2019) research exploring variation in children's social behaviors across countries as a function of parental education and family income.

Fifth, we were unable to explore the processes that explain why friendships between children with similar attributes are more stable than other friendships. Perhaps, consistent with social exchange theory, children find relationships with others similar to themselves particularly rewarding whereas differences between friends foster perceptions of inequality and increased conflicts between friends and lead to the termination of friendships (Laursen, 2017). A complementary explanation is that similarity may result if children prefer to be friends with others that are at higher or the same level on an important attribute but refrain from befriending others lower on this attribute. As shown by Shen and French (2024), this may account for the similarity in academic level in Chinese adolescents. This may also account for the homophily of low‐status children's friendships as they have limited options other than to befriend other low‐status children. To elucidate these processes, it will be useful to conduct frequent assessments of friendship, and perhaps use both quantitative and qualitative methods.

Finally, classroom friendships were assessed in the present study, but children also have friends in contexts such as neighborhoods, religious groups, and athletic teams. It is important in future studies to assess if individual attributes and dyadic similarity variables also account for the stability of friendships across contexts.

## SUMMARY AND CONCLUSIONS

The present study reveals that children's friendship stability can be predicted by both individual characteristics and similarities between friends. Children that exhibited positive traits and social skills (i.e., academic competence, high popularity and social preference) had more stable friendships than did others. Individual aggression, however, did not predict friendship stability. It is noteworthy that these predictors of friendship stability did not differ for boys and girls.

Dyadic similarity also predicted friendship stability, with significant effects in one or more countries. Social preference similarity was positively associated with friendship stability across countries, whereas others varied as a function of country differences in the reputational salience of these attributes. US, Chinese, and Indonesian children's friendship stability was respectively predicted by similarity in popularity, academic achievement, and aggression. Children maintain friendships with peers similar to themselves, but they may emphasize the attributes that are important in their culture.

These results suggest that the “reputational salience” framework (Chen & French, 2008; Hartup, 1996) may be useful for explaining the country differences in the predictors of friendships stability. Qualities appraised in a culture are more likely to impact children's friendship stability than are others (Chen & French, 2008). In the present study, we relied upon the results from empirical studies to ascertain the reputational salience of attributes. Others, however, may choose to use more general frameworks such as individualism/collectivism and the results from the World Value Survey (Haerpfer et al., 2022) to determine the reputational salience of attributes. Regardless, the evidence of cultural variations in children's friendship stability is limited and further research is needed.

## FUNDING INFORMATION

The research was partially funded by the International Collaboration Research and Publication Grant awarded to Urip Purwono by the Directorate General of Higher Education, Ministry of Education, Research, Culture, and Technology, Republic of Indonesia. This research was also partially funded by an award to Urip Purwono from the Jacobs Foundation.

## DATA AVAILABILITY STATEMENT

The data used in this study are not publically available.

## Supporting information


Data S1.

## References

[cdev14189-bib-0001] Asher, S. R. , & Coie, J. D. (1990). Peer rejection in childhood. Cambridge University Press.

[cdev14189-bib-0002] Azmitia, M. , Kamprath, N. A. , & Linnet, J. (1997). Intimacy and conflict: The dynamics of boys' and girls' friendships during middle childhood and early adolescence. In L. H. Meyer , H. S. Park , M. Grenot‐Scheyer , I. S. Schwartz , & B. Harry (Eds.), Making friends: The influences of culture and development (pp. 171–187). Brookes‐Cole.

[cdev14189-bib-0003] Bem, S. L. (1983). Gender schema theory and its implications for child development: Raising gender‐aschematic children in a gender‐schematic society. Signs, 8, 598–616. 10.1086/493998

[cdev14189-bib-0004] Benenson, J. F. , & Christakos, A. (2003). The greater fragility of females' versus males' closest same‐sex friendships. Child Development, 74, 1123–1129. 10.1111/1467-8624.00596 12938708

[cdev14189-bib-0005] Berndt, T. J. , Hawkins, J. A. , & Hoyle, S. G. (1986). Changes in friendship during a school year: Effects on children's and adolescents' impressions of friendship and sharing with friends. Child Development, 57, 1284–1297. 10.2307/1130451

[cdev14189-bib-0006] Cairns, R. B. , Leung, M. C. , Buchanan, L. , & Cairns, B. D. (1995). Friendships and social networks in childhood and adolescence: Fluidity, reliability, and interrelations. Child Development, 66, 1330–1345. 10.1111/j.1467-8624.1995.tb00938.x 7555219

[cdev14189-bib-0007] Chen, X. , & French, D. C. (2008). Children's social competence in cultural context. Annual Review of Psychology, 59, 591–616. 10.1146/annurev.psych.59.103006.093606 18154504

[cdev14189-bib-0008] Chen, X. , Rubin, K. H. , & Li, Z.‐y. (1995). Social functioning and adjustment in Chinese children: A longitudinal study. Developmental Psychology, 31, 531–539. 10.1037/0012-1649.31.4.531

[cdev14189-bib-0009] Cillessen, A. H. , Schwartz, D. , & Mayeux, L. (2011). Popularity in the peer system. Guilford Press.

[cdev14189-bib-0010] DeLay, D. , Laursen, B. , Kiuru, N. , Salmela‐Aro, K. , & Nurmi, J. E. (2013). Selecting and retaining friends on the basis of cigarette smoking similarity. Journal of Research on Adolescence, 23, 464–473. 10.1111/jora.12017

[cdev14189-bib-0011] Dijkstra, J. K. , Cillessen, A. H. N. , & Borch, C. (2013). Popularity and adolescent friendship networks: Selection and influence dynamics. Developmental Psychology, 49, 1242–1252. 10.1037/a0030098 22985296

[cdev14189-bib-0012] Dishion, T. J. , Andrews, D. W. , & Crosby, L. (1995). Antisocial boys and their friends in early adolescence: Relationship characteristics, quality, and interactional process. Child Development, 66, 139–151. 10.1111/j.1467-8624.1995.tb00861.x 7497821

[cdev14189-bib-0013] Eder, D. (1985). The cycle of popularity: Interpersonal relations among female adolescents. Sociology of Education, 58, 154–165. 10.2307/2112416

[cdev14189-bib-0014] Ellis, W. E. , & Zarbatany, L. (2007). Explaining friendship formation and friendship stability: The role of children's and friends' aggression and victimization. Merrill‐Palmer Quarterly, 53, 79–104. 10.1353/mpq.2007.0001

[cdev14189-bib-0015] Fielding, A. , & Goldstein, H. (2006). Cross‐classified and multiple membership structures in multilevel models: An introduction and review (research report no. 791). DfES.

[cdev14189-bib-0016] Flannery, K. M. , & Smith, R. L. (2017). Are peer status, friendship quality, and friendship stability equivalent markers of social competence? Adolescent Research Review, 2, 331–340. 10.1007/s40894-016-0042-z

[cdev14189-bib-0017] French, D. C. (2015). Cultural templates for child and adolescent friendships. In L. A. Jensen (Ed.), Oxford handbook of human development and culture: An interdisciplinary perspective (pp. 425–437). Oxford University Press.

[cdev14189-bib-0018] French, D. C. , Eisenberg, N. , Purwono, U. , & Sallquist, J. A. (2012). Indonesian Muslim adolescents and the ecology of religion. In G. Trommsdorff & X. Chen (Eds.), Values, religion, and culture in adolescent development (pp. 146–163). Cambridge University Press.

[cdev14189-bib-0019] French, D. C. , Jansen, E. A. , & Pidada, S. (2002). United States and Indonesian children's and adolescents' reports of relational aggression by disliked peers. Child Development, 73, 1143–1150. 10.1111/1467-8624.00463 12146739

[cdev14189-bib-0020] French, D. C. , Niu, L. , & Purwono, U. (2016). Popularity of Indonesian adolescents: Do the findings from the USA generalize to a Muslim majority developing country? Social Development, 25, 405–421. 10.1111/sode.12148

[cdev14189-bib-0021] French, D. C. , Shen, M. , & Jin, S. (2022). Are adolescent leaders prosocial and aggressive? International Journal of Behavioral Development, 46, 346–357. 10.1177/01650254221100250

[cdev14189-bib-0022] Guimond, F. A. , Laursen, B. , Hartl, A. C. , & Cillessen, A. H. (2019). Differences in internalizing symptoms anticipate adolescent friendship dissolution. Journal of Research on Adolescence, 29, 924–937. 10.1111/jora.12432 29984870

[cdev14189-bib-0023] Haerpfer, C. , Inglehart, R. , Moreno, A. , Welzel, C. , Kizilova, K. , Diez‐ Medrano, J. , Lagos, M. , Norris, P. , Ponarin, E. , & Puranen, B. (2022). World values survey: Round seven—Country‐pooled datafile version 5.0. JD Systems Institute & WVSA Secretariat. 10.14281/18241.20

[cdev14189-bib-0024] Hafen, C. A. , Laursen, B. , Burk, W. J. , Kerr, M. , & Stattin, H. (2011). Homophily in stable and unstable adolescent friendships: Similarity breeds constancy. Personality and Individual Differences, 51, 607–612. 10.1016/j.paid.2011.05.027

[cdev14189-bib-0025] Hartl, A. C. , Laursen, B. , & Cillessen, A. H. (2015). A survival analysis of adolescent friendships: The downside of dissimilarity. Psychological Science, 26, 1304–1315. 10.1177/0956797615588751 26187246 PMC4529362

[cdev14189-bib-0026] Hartup, W. W. (1996). The company they keep: Friendships and their developmental significance. Child Development, 67, 1–13. 10.2307/1131681 8605821

[cdev14189-bib-0027] Hartup, W. W. , & Stevens, N. (1997). Friendships and adaptation in the life course. Psychological Bulletin, 121, 355–370. 10.1037/0033-2909.121.3.355

[cdev14189-bib-0028] Hinde, R. A. , & Stevenson‐Hinde, J. (1976). Towards understanding relationships: Dynamic stability. In P. P. G. Bateson & R. A. Hinde (Eds.), Growing points in ethology (pp. 451–479). Cambridge Press.

[cdev14189-bib-0029] Ho, D. Y. F. (1986). Chinese patterns of socialization: A critical review. In M. H. Bond (Ed.), The psychology of the Chinese people (pp. 1–37). Oxford University Press.

[cdev14189-bib-0030] Howes, C. (1990). Social status and friendship from kindergarten to third grade. Journal of Applied Developmental Psychology, 11, 321–330. 10.1016/0193-3973(90)90013-A

[cdev14189-bib-0031] Jakobsen, J. C. , Gluud, C. , Wetterslev, J. , & Winkel, P. (2017). When and how should multiple imputation be used for handling missing data in randomized clinical trials–a practical guide with flowcharts. BMC Medical Research Methodology, 17, 1–10. 10.1186/s12874-017-0442-1 29207961 PMC5717805

[cdev14189-bib-0032] Kandel, D. B. (1978). Homophily, selection, and socialization in adolescent friendships. American Journal of Sociology, 84, 427–436.

[cdev14189-bib-0033] Kenny, D. A. , Kashy, D. A. , & Cook, W. L. (2020). Dyadic data analysis. Guilford Publications.

[cdev14189-bib-0034] Kim, J. H. (2019). Multicollinearity and misleading statistical results. Korean Journal of Anesthesiology, 72, 558–569. 10.4097/kja.19087 31304696 PMC6900425

[cdev14189-bib-0035] Kupersmidt, J. B. , DeRosier, M. E. , & Patterson, C. P. (1995). Similarity as the basis for children's friendships: The roles of sociometric status, aggressive and withdrawn behavior, academic achievement and demographic characteristics. Journal of Social and Personal Relationships, 12, 439–452. 10.1177/0265407595123007

[cdev14189-bib-0036] Laninga‐Wijnen, L. , Harakeh, Z. , Steglich, C. , Dijkstra, J. K. , Veenstra, R. , & Vollebergh, W. (2017). The norms of popular peers moderate friendship dynamics of adolescent aggression. Child Development, 88, 1265–1283. 10.1111/cdev.12650 27779756

[cdev14189-bib-0037] Lansford, J. E. , Malone, P. S. , Tapanya, S. , Tirado, L. M. U. , Zelli, A. , Alampay, L. P. , Al‐Hassan, S. M. , Bacchini, D. , Bornstein, M. H. , Chang, L. , Deater‐Deckard, K. , Giunta, L. D. , Dodge, K. A. , Oburu, P. , Pastorelli, C. , Skinner, A. T. , Sorbring, E. , & Steinberg, L. (2019). Household income predicts trajectories of child internalizing and externalizing behavior in high‐, middle‐, and low‐income countries. International Journal of Behavioral Development, 43, 74–79. 10.1177/0165025418783272 30739968 PMC6364858

[cdev14189-bib-0038] Laursen, B. (2017). Making and keeping friends: The importance of being similar. Child Development Perspectives, 11, 282–289. 10.1111/cdep.12246

[cdev14189-bib-0039] Lempers, J. D. , & Clark‐Lempers, D. S. (1993). A functional comparison of same‐sex and opposite‐sex friendships during adolescence. Journal of Adolescent Research, 8, 89–108. 10.1177/074355489381007

[cdev14189-bib-0040] Li, Y. , Xie, H. , & Shi, J. (2012). Chinese and American children's perceptions of popularity determinants: Cultural differences and behavioral correlates. International Journal of Behavioral Development, 36, 420–429. 10.1177/0165025412446393

[cdev14189-bib-0041] Logis, H. A. , Rodkin, P. C. , Gest, S. D. , & Ahn, H. J. (2013). Popularity as an organizing factor of preadolescent friendship networks: Beyond prosocial and aggressive behavior. Journal of Research on Adolescence, 23, 413–423. 10.1111/jora.12033

[cdev14189-bib-0042] Magnis‐Suseno, F. (1997). Javanese Etics and world‐view. The Javanese idea of the good life.

[cdev14189-bib-0043] Masten, A. S. , Morison, P. , & Pellegrini, D. S. (1985). A Revised Class Play method of peer assessment. Developmental Psychology, 21, 523–533. 10.1037/0012-1649.21.3.523

[cdev14189-bib-0044] McDonald, K. L. , Dashiell‐Aje, E. , Menzer, M. M. , Rubin, K. H. , Oh, W. , & Bowker, J. C. (2013). Contributions of racial and socio‐behavioral homophily to friendship stability and quality among same‐race and cross‐race friends. The Journal of Early Adolescence, 33, 897–919. 10.1177/0272431612472259

[cdev14189-bib-0045] Merten, D. E. (1997). The meaning of meanness: Popularity, competition, and conflict among junior high school girls. Sociology of Education, 70, 175–191. 10.2307/2673207

[cdev14189-bib-0046] Meter, D. J. , & Card, N. A. (2016). Stability of children's and adolescents' friendships: A meta‐analytic review. Merrill‐Palmer Quarterly, 62, 252–284. 10.13110/merrpalmquar1982.62.3.0252

[cdev14189-bib-0047] Ng‐Knight, T. , Shelton, K. H. , Riglin, L. , Frederickson, N. , McManus, I. C. , & Rice, F. (2019). ‘Best friends forever’? Friendship stability across school transition and associations with mental health and educational attainment. British Journal of Educational Psychology, 89, 585–599. 10.1111/bjep.12246 30259513

[cdev14189-bib-0048] Nielson, M. G. , Delay, D. , Flannery, K. M. , Martin, C. L. , & Hanish, L. D. (2020). Does gender‐bending help or hinder friending? The roles of gender and gender similarity in friendship dissolution. Developmental Psychology, 56, 1157–1169. 10.1037/dev0000930 32297763

[cdev14189-bib-0049] Niu, L. , Jin, S. , Li, L. , & French, D. C. (2016). Popularity and social preference in Chinese adolescents: Associations with social and behavioral adjustment. Social Development, 25, 828–845. 10.1111/sode.12172

[cdev14189-bib-0050] Oh, W. , Bowker, J. , Kim, H. , Santos, A. , Ribeiro, O. , Guedes, M. , Freitas, M. , Song, S. , & Rubin, K. H. (2021). Distinct profiles of relationships with mothers, fathers, and best friends and socio‐emotional functioning in early adolescence: A cross‐cultural study. Child Development, 92, 1154–1170. 10.1111/cdev.13610 34259345 PMC9292231

[cdev14189-bib-0051] Poulin, F. , & Chan, A. (2010). Friendship stability and change in childhood and adolescence. Developmental Review, 30, 257–272. 10.1016/j.dr.2009.01.001

[cdev14189-bib-0052] Proulx, M. F. , & Poulin, F. (2013). Stability and change in kindergartners' friendships: Examination of links with social functioning. Social Development, 22, 111–125. 10.1111/sode.12001

[cdev14189-bib-0053] Rambaran, J. A. , Hopmeyer, A. , Schwartz, D. , Steglich, C. , Badaly, D. , & Veenstra, R. (2017). Academic functioning and peer influences: A short‐term longitudinal study of network–behavior dynamics in middle adolescence. Child Development, 88, 523–543. 10.1111/cdev.12611 27580016

[cdev14189-bib-0054] Rose, A. J. , & Smith, R. L. (2018). Gender and peer relationships. In W. M. Bukowski , B. Laursen , & K. H. Rubin (Eds.), Handbook of peer interactions, relationships, and groups (pp. 571–589). Guilford Publications.

[cdev14189-bib-0055] Rubin, K. H. , Oh, W. , Menzer, M. , & Ellison, K. (2011). Dyadic relationships from a cross cultural perspective: Parent‐child relationships and friendship. In X. Chen & K. H. Rubin (Eds.), Socioemotional development in cultural context (pp. 208–236). The Guilford Press.

[cdev14189-bib-0056] Shen, M. , & French, D. C. (2024). Peer relationships and Chinese adolescents' academic achievement: Selection and influence. American Educational Research Journal, 61, 177–207. 10.3102/00028312231208675

[cdev14189-bib-0057] van den Berg, Y. H. , Lansu, T. A. , & Cillessen, A. H. (2020). Preference and popularity as distinct forms of status: A meta‐analytic review of 20 years of research. Journal of Adolescence, 84, 78–95. 10.1016/j.adolescence.2020.07.010 32891019

[cdev14189-bib-0058] van Workum, N. , Scholte, R. H. , Cillessen, A. H. , Lodder, G. M. , & Giletta, M. (2013). Selection, deselection, and socialization processes of happiness in adolescent friendship networks. Journal of Research on Adolescence, 23, 563–573. 10.1111/jora.12035

[cdev14189-bib-0059] Yue, X. , & Zhang, Q. (2023). The association between peer rejection and aggression types: A meta‐analysis. Child Abuse & Neglect, 135, 105974. 10.1016/j.chiabu.2022.105974 36521401

[cdev14189-bib-0061] Zhang, K. , Duan, J. , Gest, S. , Chen, X. , Liu, J. , Li, D. , & French, D. C. (2024). Peer relationships mediate the pathways from behavioral qualities to United States and Chinese children's loneliness. Child Development, 95, e21–e34. 10.1111/cdev.13982. 37561124

[cdev14189-bib-0060] Zhang, X. , Pomerantz, E. M. , Qin, L. , Logis, H. , Ryan, A. M. , & Wang, M. (2018). Characteristics of likability, perceived popularity, and admiration in the early adolescent peer system in the United States and China. Developmental Psychology, 54, 1568–1581. 10.1037/dev0000544 30047777

