# Supplementary material for: Children's friendship stability in the United States, China, and Indonesia: Associations with individual attributes and dyadic similarity
Source: Child Dev. 2024 Oct 26;96(2):591–605. doi: 10.1111/cdev.14189 (PMC11868691; doi:10.1111/cdev.14189)
Supplement: Supplementary file 1 — Data S1. [file CDEV-96-591-s001.docx]

**Supplementary Materials**

**Cross-Classified Multilevel Modeling**

Fielding and Goldstein (2006) introduced an extension of multilevel analysis, i.e., the cross-classified multilevel modeling, to address the complex data structure in real world cases. For example, siblings from the same families are nested within their parents. Thus, to study parental effects on siblings’ development, the multilevel analysis is preferred as this accounts for the interdependence of multiple siblings in terms of parental effects. However, the data structure becomes complicated if one attempts to explore the maternal and paternal influence separately. Multiple siblings are simultaneously nested within their mothers *and* their fathers. In this case, each child is no longer nested within one higher unit (e.g., the family or the combination of mothers and fathers) but within two (i.e., the mothers *and* the fathers). The conventional multilevel analysis is insufficient to address this data structure because it does not lower units to be across classified within multiple higher units.

The cross-classified multilevel analysis allows lower units to simultaneously belong to two or more higher units. Consequently, the effects of multiple higher units on the lower units can be examined in one analysis. For example, friendships have a cross-classified data structure. Six possible friendships can emerge between Child A, B, and C (i.e., A-B, A-C, B-A, B-C, C-A, and C-B). Dyad A-B and A-C are nested within Child A; Dyad B-A and B-C belong to Child B. Each friend dyad is entered twice (e.g., A-B and B-A) because friend dyads are indistinguishable; children need to be both focal children and friends of others (Kenney et al. 2020). In this friendship case, Child A is the focal child in Dyad A-B with Child B as the friend; Child B is the focal child in Dyad B-A in which Child A acts as the friend.

The cross-classified structure of friend dyads come from that each friendship has two partners and some children shared common friends. Child A and B are respectively the focal children for Dyad A-C and B-C; Child C is the common friend for these two dyads. Consequently, in addition to being nested within their focal children (i.e., Child A and B), Dyad A-C and Dyad B-C are also nested within the common friend Child C. It is worth noting that Child C acts as the *friend* in these two dyads. Child C is the focal child in Dyad C-A and C-B.

The reason why we distinguished focal children and friends lies on that focal children and their friends individually contribute to friendship duration. For example, Child C may exhibit high levels of aggression and Child A and B decide to terminate their friendships with Child C. In this case, Child C loses two friendships (i.e., Dyad C-A and C-B) and Child A and B also respectively loses one friendship (i.e., Dyad A-C and Dyad B-C). Child C’s aggressiveness may explain the dissolution of Dyad C-A and C-B, but the termination of Dyad A-C and Dyad B-C are not attributable to the aggressiveness of Child A and B but to that of Child C. Therefore, to identify the extent to which Child A and B contributes respectively to the duration of Dyad A-C and Dyad B-C, the effects of Child C should be controlled.

In the present study, we first examined the associations between dyadic similarity variables and friendship stability at level 1, friendship level. Then, we examined the extent to which child individual attributes predicted the stabilities of their multiple friendships (e.g., A’s aggression 🡪 the stabilities of A-B and A-C) while controlling for friends’ attributes (e.g., C’s aggression🡪 the stabilities of A-C) at level 2, child level. Two random effects were estimated because as noted earlier, the variations of friendship stability at the child level are attributable to two parts: Friendships of some children may dissolve faster than others (i.e., random effects for focal children) and friendships that include some children as friends may end more rapidly than others (i.e., random effects for friends).

It is worth noting that the estimations of either fixed or random effects for focal children and their friends are identical. This is because friend dyads were doubly entered (e.g., Child A’s contribution to Dyad A-B in which Child A is the focal child = Child A’s contribution to Dyad B-A in which Child A is the friend).

**Cross-Country Analyses: Model Equations and Establishment Procedures**

Four models were established respectively for social preference, popularity, aggression, academic achievement. The procedures and equations were the same for each model. Dyadic similarity variables and individual attributes were centered within clusters (i.e., country groups) using within-country standardization.

**Unconditional Model.** Following the procedures outlined in Nielson et al.’s (2020) study, we first established an unconditional model with no predictor included. The equations were listed below.

Level 1:

*Friendship Stability _j(i1,i2)_* = *β*_00(_*_i1,i2)_ + e* *_j(i1,i2)_*

Level 2:

*β*_00_*_(i1,i2)_* =*γ*_000_*_(i1,i2)_* + *μ*_000_*_i1_* + *μ*_000_*_i2_*

The notation *j* refers to the specific friend dyad; *i1* and *i2* respectively refers to the focal child and the friend of the focal child. The “*j(i1,i2)*” indicates the friendship *j* of focal child *i1* and the friend *i2*. Two random effects, *μ*_000_*_i1_* and *μ*_000_*_i2_*, were estimated. The first one indicated the random effects due to between-focal-child (*i1*) differences and the later one refers to the random effects due to between-friend (*i2*) differences. Because the focus of the present study is only on the focal children, the unconditional ICC was computed as *σ^2^μ*_000_*_i1_*/(*σ^2^μ*_000_*_i1_*+*σ^2^μ*_000_*_i2_*+*σ^2^ e _j(i1,i2)_*) (i.e., the proportion of between-focal-child differences variances in the overall variances of friendship stability).

**Model 1.** Based on the unconditional model, Model 1 was built by including control variables. Control variables included sex, focal children’s number of friends, friends’ number of friends, friends’ attribute (e.g., friend popularity), and country groups that were dummy coded. The example below used the Chinese sample as the reference group. To switch the reference group to the US sample, the two countries dummy variables were replaced with “Country: China vs US” and “Country: Indonesia vs US”.

Level 1:

*Friendship Stability _j(i1,i2)_* = *β*_00(_*_i1,i2)_ + e _j(i1,i2)_*

Level 2:

*β*_00_ *_(i1,i2)_* =*γ*_000_ *_(i1,i2)_* + *γ*_001_*_i1_* (Sex) + *γ*_002_*_i1_* (Number of Friends) + *γ*_003_*_i2_* (Friends’ Number of Friends) + *γ*_004_*_i2_* (Friend Attribute) + *γ*_005_*_i1_* (Country: US vs China) + *γ*_006_*_i1_* (Country: Indonesia vs China) + *μ*_000_*_i1_* + *μ*_000_*_i2_*

**Model 2**. This was established based on Model 1 with friendship-level predictors (i.e., dyadic similarity variable) included.

Level 1:

*Friendship Stability _j(i1,i2)_* = *β*_00_*_(i1,i2)_* + *β*_01_*_(i1,i2)_* (Dyadic Similarity) *+ e _j(i1,i2)_*

Level 2:

*β*_00_ *_(i1,i2)_* =*γ*_000_ *_(i1,i2)_* + *γ*_001_*_i1_* (Sex) + *γ*_002_*_i1_* (Number of Friends) + *γ*_003_*_i2_* (Friends’ Number of Friends) + *γ*_004_*_i2_* (Friend Attribute) + *γ*_005_*_i1_* (Country: US vs China) + *γ*_006_*_i1_* (Country: Indonesia vs China) + *μ*_000_*_i1_* + *μ*_000_*_i2_*

*β*_01_*_(i1,i2)_* = *γ*_100_ *_(i1,i2)_*

**Model 3.** Model 3 included the individual attribute. Model 3 assessed the main effects of the dyadic similarity and the individual attribute on friendship stability.

Level 1:

*Friendship Stability _j(i1,i2)_* = *β*_00_*_(i1,i2)_* + *β*_01_*_(i1,i2)_* (Dyadic Similarity) *+ e _j(i1,i2)_*

Level 2:

*β*_00_ *_(i1,i2)_* =*γ*_000_ *_(i1,i2)_* + *γ*_001_*_i1_* (Sex) + *γ*_002_*_i1_* (Number of Friends) + *γ*_003_*_i2_* (Friends’ Number of Friends) + *γ*_004_*_i2_* (Friend Attribute) + *γ*_005_*_i1_* (Country: US vs China) + *γ*_006_*_i1_* (Country: Indonesia vs China) + *γ*_007_*_i1_* (Individual Attribute)+*μ*_000_*_i1_* + *μ*_000_*_i2_*

*β*_01_*_(i1,i2)_* = *γ*_100_ *_(i1,i2)_*

**Model 4.** We examined two-way interactions in Model 4, including sex by dyadic similarity variable, sex by individual attribute, country by dyadic similarity variable, and country by individual attribute. Non-significant interactions were excluded from the model.

Level 1:

*Friendship Stability _j(i1,i2)_* = *β*_00_*_(i1,i2)_* + *β*_01_*_(i1,i2)_* (Dyadic Similarity) *+ e _j(i1,i2)_*

Level 2:

*β*_00_ *_(i1,i2)_* =*γ*_000_ *_(i1,i2)_* + *γ*_001_*_i1_* (Sex) + *γ*_002_*_i1_* (Number of Friends) + *γ*_003_*_i2_* (Friends’ Number of Friends) + *γ*_004_*_i2_* (Friend Attribute) + *γ*_005_*_i1_* (Country: US vs China) + *γ*_006_*_i1_* (Country: Indonesia vs China) + *γ*_007_*_i1_* (Individual Attribute) + *γ*_008_*_i1_* (Individual Attribute × Sex) + *γ*_009_*_i1_* (Individual Attribute × Country: US vs China) + *γ*_010_*_i1_* (Individual Attribute × Country: Indonesia vs China) + *μ*_000_*_i1_* + *μ*_000_*_i2_*

*β*_01_*_(i1,i2)_* = *γ*_100_ *_(i1,i2)_*+ *γ*_101_*_i1_* (Sex) + *γ*_102_*_i1_* (Country: US vs China) + *γ*_103_*_i1_* (Country: Indonesia vs China)

Table S1

*Correlations Between Variables for the US Sample*

|  | 1 | 2 | 3 | 4 | 5 | 6 | 7 | 8 | 9 | 10 |
| --- | --- | --- | --- | --- | --- | --- | --- | --- | --- | --- |
| 1.Friendship stability |  | 0.04 | 0.03 | 0.06^*^ | -0.07^*^ | 0.13^**^ | 0.10^**^ | 0.01 | 0.05 | 0.15^**^ |
| 2.Popularity similarity | -0.05 |  | 0.41^**^ | 0.11^**^ | 0.01 | -0.08^**^ | -0.07^*^ | 0.03 | -0.05 | -0.14^**^ |
| 3.Preference similarity | 0.09^**^ | 0.26^**^ |  | 0.07^*^ | 0.14^**^ | 0.13^**^ | 0.17^**^ | 0.03 | 0.00 | -0.05 |
| 4.Aggression similarity | 0.00 | 0.10^**^ | 0.20^**^ |  | -0.03 | -0.10^**^ | 0.13^**^ | -0.49^**^ | 0.15^**^ | 0.13^**^ |
| 5.Academic similarity | 0.02 | 0.09^**^ | 0.11^**^ | -0.04 |  | 0.01 | 0.04 | 0.05 | -0.07^*^ | 0.00 |
| 6.Individual/Friend popularity | 0.16^**^ | -0.02 | 0.19^**^ | -0.16^**^ | 0.05 |  | 0.67^**^ | 0.28^**^ | 0.19^**^ | 0.21^**^ |
| 7.Individual/Friend preference | 0.07^*^ | 0.03 | 0.18^**^ | 0.11^**^ | -0.05 | 0.43^**^ |  | -0.21^**^ | 0.30^**^ | 0.40^**^ |
| 8.Individual/Friend aggression | 0.02 | 0.00 | -0.01 | -0.46^**^ | 0.04 | 0.25^**^ | -0.38^**^ |  | -0.30^**^ | -0.20^**^ |
| 9.Individual/Friend academic | 0.08^*^ | 0.03 | 0.07^*^ | 0.05 | -0.02 | 0.01 | 0.19^**^ | -0.15^**^ |  | 0.21^**^ |
| 10.Number of friends | 0.06^*^ | -0.08^**^ | 0.04 | -0.11^**^ | -0.03 | 0.16^**^ | 0.39^**^ | -0.09^**^ | 0.06^*^ |  |

*Note.* Lower diagonal = girls; Upper diagonal = boys. Samples included 1026 girls’ and 1018 boys’ friendship dyads.

**p*<.05; ***p*<.01; ****p*<.001.

Table S2

*Correlations Between Variables for the Chinese Sample*

|  | 1 | 2 | 3 | 4 | 5 | 6 | 7 | 8 | 9 | 10 |
| --- | --- | --- | --- | --- | --- | --- | --- | --- | --- | --- |
| 1.Friendship stability |  | 0.10^**^ | 0.09^**^ | 0.02 | 0.13^**^ | 0.11^**^ | 0.03 | -0.03 | 0.09^**^ | -0.16^**^ |
| 2.Popularity similarity | -0.02 |  | -0.04 | -0.06^*^ | -0.05 | 0.58^**^ | 0.14^**^ | 0.03 | 0.21^**^ | 0.03 |
| 3.Preference similarity | 0.03 | -0.29^**^ |  | 0.56^**^ | 0.21^**^ | 0.00 | 0.39^**^ | -0.35^**^ | 0.22^**^ | 0.04 |
| 4.Aggression similarity | 0.00 | -0.06^*^ | 0.49^**^ |  | 0.11^**^ | -0.07^*^ | 0.35^**^ | -0.60^**^ | 0.17^**^ | -0.03 |
| 5.Academic similarity | 0.10^**^ | -0.03 | 0.17^**^ | 0.03 |  | 0.04 | 0.17^**^ | -0.12^**^ | 0.32^**^ | 0.03 |
| 6.Individual/Friend popularity | 0.04 | 0.50^**^ | -0.14^**^ | -0.10^**^ | 0.11^**^ |  | 0.18^**^ | 0.11^**^ | 0.35^**^ | 0.04 |
| 7.Individual/Friend preference | 0.06^*^ | 0.24^**^ | 0.21^**^ | 0.24^**^ | 0.18^**^ | 0.52^**^ |  | -0.63^**^ | 0.49^**^ | 0.29^**^ |
| 8.Individual/Friend aggression | -0.03 | 0.02 | -0.33^**^ | -0.61^**^ | -0.04 | 0.07^*^ | -0.42^**^ |  | -0.23^**^ | -0.02 |
| 9.Individual/Friend academic | 0.07^*^ | 0.19^**^ | 0.08^**^ | 0.01 | 0.39^**^ | 0.44^**^ | 0.42^**^ | -0.03 |  | 0.06 |
| 10.Number of friends | 0.02 | 0.25^**^ | 0.00 | 0.04 | 0.03 | 0.40^**^ | 0.45^**^ | -0.12^**^ | 0.21^**^ |  |

*Note.* Lower diagonal = girls; Upper diagonal = boys. Samples included 1046 girls’ and 906 boys’ friendship dyads.

**p*<.05; ***p*<.01; ****p*<.001.

Table S3

*Correlations Between Variables for the Indonesian Sample*

|  | 1 | 2 | 3 | 4 | 5 | 6 | 7 | 8 | 9 | 10 |
| --- | --- | --- | --- | --- | --- | --- | --- | --- | --- | --- |
| 1.Friendship stability |  | 0.01 | 0.10^**^ | 0.15^**^ | 0.00 | 0.06 | 0.15^**^ | -0.08^*^ | 0.03 | -0.02 |
| 2.Popularity similarity | -0.09^*^ |  | 0.17^**^ | 0.05 | 0.15^**^ | -0.47^**^ | -0.22^**^ | -0.03 | -0.17^**^ | -0.08^**^ |
| 3.Preference similarity | 0.10^*^ | -0.02 |  | 0.31^**^ | 0.12^**^ | 0.01 | 0.22^**^ | -0.17^**^ | 0.09^**^ | 0.04 |
| 4.Aggression similarity | 0.11^**^ | 0.05 | 0.60^**^ |  | 0.02 | 0.00 | 0.23^**^ | -0.52^**^ | 0.15^**^ | -0.11^**^ |
| 5.Academic similarity | 0.08^*^ | 0.23^**^ | 0.18^**^ | 0.15^**^ |  | -0.05 | -0.03 | -0.04 | -0.07^*^ | -0.04 |
| 6.Individual/Friend popularity | 0.04 | -0.43^**^ | 0.01 | -0.06 | -0.12^**^ |  | 0.44^**^ | 0.07^*^ | 0.34^**^ | 0.10^**^ |
| 7.Individual/Friend preference | 0.09^*^ | -0.16^**^ | 0.20^**^ | 0.27^**^ | -0.03 | 0.26^**^ |  | -0.43^**^ | 0.36^**^ | 0.16^**^ |
| 8.Individual/Friend aggression | -0.08^*^ | 0.01 | -0.35^**^ | -0.55^**^ | -0.02 | 0.13^**^ | -0.49^**^ |  | -0.29^**^ | 0.16^**^ |
| 9.Individual/Friend academic | 0.03 | -0.14^**^ | 0.12^**^ | 0.06 | -0.14^**^ | 0.32^**^ | 0.30^**^ | -0.07 |  | -0.10^**^ |
| 10.Number of friends | 0.03 | -0.10^*^ | 0.04 | 0.04 | 0.03 | 0.24^**^ | 0.34^**^ | -0.05 | 0.07 |  |

*Note.* Lower diagonal = girls; Upper diagonal = boys. Samples included 502 girls’ and 790 boys’ friendship dyads.

**p*<.05; ***p*<.01; ****p*<.001.

Table S4

*Model Fit Indices and Results for Likelihood Ratio Tests for Cross-Culture Models*

|  | *df* | AIC | BIC | -2LL | *LRT* |
| --- | --- | --- | --- | --- | --- |
| Model 0: Unconditional model | 4 | 6895.1 | 6921.4 | 6887.0 | - |
|  |  |  |  |  |  |
| Popularity Model |  |  |  |  |  |
| Model 1: Control variables | 10 | 6828.6 | 6894.3 | 6808.6 | 78.51^***^ |
| Model 2: Popularity similarity | 11 | 6830.4 | 6902.7 | 6808.4 | 0.12 |
| Model 3: Individual popularity | 12 | 6814.8 | 6893.6 | 6790.8 | 17.69^***^ |
| Model 4: Significant interactions | 14 | 6810.1 | 6902.1 | 6782.1 | 8.65^*^ |
|  |  |  |  |  |  |
| Social Preference Model |  |  |  |  |  |
| Model 1: Control variables | 10 | 6822.5 | 6888.3 | 6802.6 | 84.54^***^ |
| Model 2: Rejection similarity | 11 | 6807.3 | 6879.6 | 6785.2 | 17.25^***^ |
| Model 3: Individual preference | 12 | 6794.3 | 6873.2 | 6770.4 | 14.96^***^ |
|  |  |  |  |  |  |
| Aggression Model |  |  |  |  |  |
| Model 1: Control variables | 10 | 6847.8 | 6913.5 | 6827.8 | 59.28^***^ |
| Model 2: Aggression similarity | 11 | 6833.4 | 6905.7 | 6811.4 | 16.39^***^ |
| Model 3: Individual aggression | 12 | 6835.3 | 6914.2 | 6811.3 | 0.05 |
| Model 4: Significant interactions | 14 | 6824.4 | 6916.4 | 6796.4 | 14.97^***^ |
| Academic Achievement Model | *F*-value | Δ*df* | *p* | RIV |  |
| Model 1: Control variables | 10.69 | 6 | <.001 | 0.03 |  |
| Model 2: Academic similarity | 10.18 | 1 | 0.001 | 0.08 |  |
| Model 3: Individual academic | 9.21 | 1 | 0.002 | 0.11 |  |
| Model 4: Significant interactions | 2.38 | 7 | 0.020 | 0.07 |  |

*Note.* *df* = degrees of freedom; AIC = Akaike Information Criterion; BIC = Bayesian Information Criterion; -2LL = -2loglikelihood; *LRT* = likelihood ratio tests; RIV = relative increase in variance due to nonresponse. *LRT*s were based on the comparisons between Model *k* and Model *k-1*. Because the academic achievement model was estimated using imputed data, *LRT*s was pooled from multiple date sets and other model fit indices were not available. Model 0 was the baseline model for all other models.

**p*<.05; ***p*<.01; ****p*<.001.

**References for Supplementary Materials**

Fielding, A., & Goldstein, H. (2006). *Cross-classified and multiple membership structures in multilevel models: An introduction and review* (Research Report No. 791). DfES, London, UK.

Kenny, D. A., Kashy, D. A., & Cook, W. L. (2020). *Dyadic data analysis*. Guilford Publications.

Nielson, M. G., Delay, D., Flannery, K. M., Martin, C. L., & Hanish, L. D. (2020). Does gender-bending help or hinder friending? The roles of gender and gender similarity in friendship dissolution. *Developmental Psychology, 56*, 1157–1169. [https://doi.org/10.1037/dev0000930](https://psycnet.apa.org/doi/10.1037/dev0000930)
